# Chlorhexidine gluconate 0.2% as a treatment for recalcitrant fungal keratitis in Uganda: a pilot study

**DOI:** 10.1136/bmjophth-2020-000698

**Published:** 2021-07-05

**Authors:** Simon Arunga, Tumu Mbarak, Abel Ebong, James Mwesigye, Dan Kuguminkiriza, Abeer H A Mohamed-Ahmed, Jeremy John Hoffman, Astrid Leck, Victor Hu, Matthew Burton

**Affiliations:** 1 Department of Clinical Research, London School of Hygiene and Tropical Medicine, London, UK; 2 Ophthalmology, Mbarara University of Science and Technology, Mbarara, Uganda; 3 Ruharo Eye Centre, Ruharo Mission Hospital, Mbarara, Uganda; 4 St Paul's Eye Unit, Liverpool, UK

**Keywords:** cornea, public health, infection, microbiology, epidemiology

## Abstract

**Objective:**

Fungal keratitis is a major ophthalmic public health problem, particularly in low-income and middle-income countries. The options for treating fungal keratitis are limited. Our study aimed to describe the outcomes of using chlorhexidine 0.2% eye-drops as additional treatment in the management of patients with recalcitrant fungal keratitis.

**Methods:**

This study was nested within a large cohort study of people presenting with microbial keratitis in Uganda. We enrolled patients with recalcitrant fungal keratitis not improving with topical natamycin 5% and commenced chlorhexidine 0.2%. Follow-up was scheduled for 3 months and 1 year. The main outcome measures were healing, visual acuity and scar size at final follow-up.

**Results:**

Thirteen patients were followed in this substudy. The patients were aged 27–73 years (median 43 years). Filamentous fungi were identified by microscopy of corneal scrape samples in all cases. Isolated organisms included *Aspergillus* spp, *Fusarium* spp, *Candida* spp, *Bipolaris* spp *and Acremoninum* spp. At the final follow-up, nine patients (75%) had healed; three had vision of better than 6/18. Three patients lost their eyes due to infection. In the remaining nine cases, corneal scarring was variable ranging from 4.6 to 9.4 mm (median 6.6 mm, IQR 5.9–8.0 mm); of these five had dense scars, three had moderate scars and one had a mild scar. None of the patients demonstrated signs of chlorhexidine toxicity during the follow-up.

**Conclusion:**

Chlorhexidine 0.2% was found to be a useful sequential adjunctive topical antifungal in cases of fungal keratitis not responding to natamycin 5%, which warrants further evaluation.

**Key messages**

**What is already known about this subject?**

- Microbial keratitis (MK) is a major ophthalmic public health problem, particularly in low-income and middle-income countries.
- MK is a major cause of blindness in sub-Saharan Africa.
- A large proportion of MK is due to fungal keratitis.
- Natamycin 5% eye-drops is the preferred first-line treatment option for fungal keratitis, however, not all cases respond well to natamycin.
- Other options for treatment of fungal keratitis are limited.

**What are the new findings?**

- Patients with recalcitrant fungal keratitis not responding to natamycin 5% eye-drops responded well to an additional chlorhexidine 0.2% eye-drops.

**How might these results change the focus of research or clinical practice?**

- Chlorhexidine 0.2% eye-drops could be a viable sequential combination option for patients with recalcitrant fungal keratitis not responding to natamycin 5%.

## Background

Microbial keratitis (MK) has been described as a ‘silent epidemic’, which leads to substantial ocular morbidity, with sight loss, pain and stigma.^1^ It frequently leads to dense corneal scarring, or even loss of the eye, especially when the infection is severe and/or appropriate treatment is delayed. A good outcome depends on early appropriate treatment, supported by correct identification of the causative organism, and careful follow-up.^2 3^ In low-income and middle-income countries, diagnostic and treatment resources are often not readily available and outcomes tend to be poor.^4^

Keratitis can be caused by bacteria, fungi, viruses and protozoa. Globally, the incidence of fungal keratitis (FK) is currently estimated to be more than one million cases per year.^5^ In tropical and subtropical regions FK makes up a substantial proportion of cases. For example, our previous work in Uganda found that the majority of MK presenting in a hospital setting in this country is caused by filamentous fungi.^6^ Compared with other infections, patients with FK were more likely to have a worse outcome.

The current best evidence indicates that topical natamycin 5% is the treatment of choice for filamentous FK.^7^ However, probably in common with all ocular antifungal agents, it does not appear to be effective at controlling the infection in all cases.^8 9^ In addition, natamycin is currently not readily available in many countries in sub-Saharan Africa (SSA) and it is relatively expensive. Chlorhexidine 0.2% is a widely used antiseptic agent. Chlorhexidine has been used in ophthalmology for over 30 years as an eye-drop preservative and for sterilising contact lenses, and has also been used to treat *Acanthamoeba* and FK.^10–15^ Two pilot trials comparing chlorhexidine to natamycin were suggestive of comparable or possibly greater efficacy than natamycin, and that chlorhexidine 0.2% w/v (aqueous solution) is safe to use.^11 12^ Recent studies of the susceptibility patterns of *Fusarium* spp isolates from patients with FK in the Netherlands and Tanzania indicated very good fungicidal activity.^16^

This study, from Uganda, investigated the use of chlorhexidine 0.2% w/v (aqueous solution) as a sequential combination treatment in a cohort of patients with FK who had evidence of progressive disease despite intensive topical natamycin. Outcome data up to 1 year are presented.

## Methods

This study is a subgroup of a previously reported cohort of 313 people with MK presenting to Mbarara University and Referral Hospital Eye Centre (MURHEC) and Ruharo Eye Hospitals (REH) in Mbarara, Uganda.^6^

### Patient and public involvement

Patients or the public were not involved in the design, or conduct, or reporting, or dissemination plans of our research.

### Study participants

MK was defined as loss of corneal epithelium (≥1 mm diameter) with underlying stromal infiltrate, associated with one or more signs of inflammation (conjunctival hyperaemia, anterior chamber inflammatory cells, ±hypopyon).^9^ We also included patients presenting with a deep corneal abscess (≥1 mm), defined as having all the features of MK, but without an epithelial defect. We excluded those not willing to participate, those not willing to return for follow-up, pregnant women, lactating mothers and those aged below 18 years.

### Clinical assessment

We documented participant’s basic demographic information and ophthalmic history. This included the circumstances in which their eye became infected, predisposing factors, treatment received and their ‘healthcare journey’ before reaching the eye hospital. Presenting logarithm of minimum angle of resolution visual acuity at 2 m in a dark room was measured using Peek Acuity software.^17^ Participants were examined with a slit lamp to assess the anterior segment using a structured protocol, including eyelid assessment, corneal ulcer features, anterior chamber (flare, cells, hypopyon shape and size) and perforation status. Infiltrate size was determined from the greatest diameter of the infiltrate (major axis) and the widest perpendicular diameter (minor axis).^9^ The final infiltrate size was then derived as the geometric mean of these two diameters.^9^ The same was repeated after fluorescein staining of the ulcer to determine epithelial defect sizes. High-resolution digital photographs with and without fluorescein staining were taken with a Nikon SLR 7200 digital camera with Macro lens.

### Laboratory assessment

Corneal scrape specimens were collected from the ulcer at a slit lamp or an operating microscope, using 21G needles after application of proxymetacaine anaesthetic eye-drops 0.5% (Minims Bausch & Lomb). Samples were assessed using Gram stain, potassium hydroxide and calcofluor white (CFW) preparations and direct inoculation on culture media (blood agar, chocolate agar (HBA), potato dextrose agar (PDA) and brain heart infusion broth). The number of corneal samples were dependent on how much material could be safely scraped from the cornea. The order of collection was microscopy, solid and then liquid phase media. Media were inoculated in the clinic then transported directly to the Mbarara University Department of Microbiology laboratory for processing. Media were incubated at 35°C–37°C for bacteria for up to 7 days and at 25°C for up to 21 days for fungi and observed daily. CFW preparations were examined immediately in the side lab at MURHEC using a fluorescence microscope (Zeiss Primostar ILED) by the attending ophthalmologist.

We followed a previously described approach for reporting positive microbiology results.^18^ Briefly, bacteria were identified using routine biochemical tests. Identification of fungi was according to the macroscopic appearance of cultures on PDA and microscopic appearance of conidia and spore-bearing structures. Positive culture was growth at the site of inoculation or growth on one solid medium consistent with microscopy; or semiconfluent growth at the site of inoculation on one solid medium (if bacteria); or growth of the same organism on repeated scraping. If, by microscopy, hyphae were observed in corneal tissue, but failed to grow in culture, the causative organism was reported as fungal.

A random blood sugar test and HIV counselling and testing were offered, as per the Uganda Ministry of Health HIV testing protocol. For those who were confirmed as HIV positive they were referred to the HIV care centre, which is on the hospital site.

### Treatment and follow-up

Patients were treated empirically at presentation and the treatment choice was reviewed when the microbiology results became available. Patients with FK were treated with natamycin 5% eye-drops (Zonat Sunways India), those with bacterial keratitis were treated with ofloxacin 0.3% eye-drops (Biomedica Remedies-India). Patients with fungal infection were treated hourly day and night for the first 3 days and then hourly while the patient was awake for 2 weeks. This was changed to 2 hourly for another 2 weeks and then tapered to four times a day until healed. For bacterial infections, patients were treated hourly day and night for the first 3 days and then reduced to six times a day for a further week. All patients with fungal MK were also given ofloxacin 0.3% eye-drops four times a day as prophylaxis until all epithelial defects were healed. In addition, those in pain were treated with atropine 1% eye-drops (locally formulated) and oral paracetamol tablets. Raised intraocular pressure was treated with Timolol 0.5% eye-drops (locally formulated). Those with presumed viral keratitis were treated with acyclovir 3% eye ointment (CIPLA India) five times a day for 3 weeks. Most patients were admitted during the first week.

After the initial assessment, patients were scheduled to be reassessed on at least day 2, day 7, day 21 and 3 months, and where possible we also conducted a 1-year assessment. Additional assessments were conducted as clinically indicated.

Patients with FK who were not improving were given topical chlorhexidine gluconate 0.2% as a sequential combination treatment option, in addition to the natamycin 5%. The decision to add chlorhexidine was based on the clinical judgement of the ophthalmologist at the day 7 assessment. Indications included increasing epithelial defect, increasing infiltrate size and opacity, hypopyon height, increasing inflammation (redness and pain), limbal involvement and impending perforation. The chlorhexidine 0.2% w/v (aqueous solution) we used was locally reconstituted from 20% chlorhexidine at our facility. The facility has a production unit for compounding eye medicine and is certified by the Uganda National Drug Authority. Patients on chlorhexidine were treated hourly while the patient was awake for 2 weeks. This was changed to 2 hourly for another 2 weeks and then tapered to four times a day until healed.

The main outcome measures were final best-corrected vision at 3 months, blindness (<3/60 in the affected eye) at 3 months and loss of the eye at 3 months. Scar density was also graded as ‘no scar’ (clear cornea), ‘mild scar’ (anterior chamber structures clearly visible through the scar), ‘moderate scar’ (anterior chamber structures vaguely visible through the scar) and ‘dense scar’ (anterior chamber structures completely obscured by the scar).

## Results

A total of 313 patients with MK were enrolled in the study; 168 (54%) had FK. Of these, 13 people with FK had clinical evidence of deterioration while on intensive topical natamycin. The demographic and clinical characteristics of the 13 participants are presented in table 1. Eight (62%%) were male. Their ages ranged between 27 and 73 years (median 43 years, IQR 38–57 years). Seven reported their main occupation to be farming. We were able to follow up seven patients to 1 year and three additional patients to 3 months although one had to be eviscerated at the end of the 3 months follow-up. One patient was lost to follow-up after day 45. Two people had such a severe infection that it required evisceration at day 14. Out of the 13 participants, one person was diabetic on insulin and four people were HIV positive (one previously undiagnosed).

**Table 1:** Demographic and clinical characteristics at presentation and final outcomes

Case	Occupation	Organism	Diabetes	HIV	Presenting visual acuity	Presenting infiltrate size (geometric mean)	Perforation at presentation	Duration of follow-up	Final visual acuity	Outcome	Corneal clarity at final visit (ulcer site)	Final scar size (mm)	Remnant corneal clarity
1	Farmer	*Aspergillius* spp	No	Pos	6/36	2.59	No	1 year	6/18	Healed	Dense scar	6.1	Clear
2	Civil servant	*Aspergillius* spp	No	Pos	6/6	4.24	No	45 days	N/A	Lost to follow-up	N/A	4.6	N/A
3	Other	No growth	No	Neg	6/36	8.49	No	1 year	6/6	Healed	Mild scar	6.4*	Clear
4	Self-employed	*Fusarium* spp	No	Neg	PL	7.32	Yes†	14 days	NPL	Eviscerated	Not applicable	–	N/A
5	Farmer	*Bipolaris* spp	Yes	Neg	HM	8.28	No	1 year	1/60	Healed	Moderate scar	5.8	Clear
6	Farmer	No growth	No	Neg	2/60	7.10	No	1 year	6/18	Healed	Dense scar	8.0	Clear
7	Farmer	Unidentified	No	Neg	PL	8.94	No	1 year	PL	Healed	Dense scar	8.0	Clear
8	None	*Fusarium* spp	No	Neg	4/60	4.40	No	1 year	6/36	Healed	Moderate scar	5.0	Clear
9	Self-employed	*Candida* spp	No	Pos	HM	1.73	No	14 days	NPL	Eviscerated	Not applicable	–	N/A
10	Farmer	*Acremonium* spp	No	Neg	HM	9.09	No	90 days	HM	Healed	Dense scar	8.8	Clear
11	Farmer	*Lasiodiplodia theobromae*	No	Pos	HM	7.08	No	90 days	2/60	Healed	Dense scar	6.8	Clear
12	Farmer	*Bipolaris* spp	No	Neg	HM	6.88	No	1 year	HM	Healed	Moderate scar	9.4*	Mildly Hazy
13	Self-employed	*Fusarium* spp	No	Neg	4/60	7.60	No	90 days	NPL	Eviscerated	Not applicable	–	N/A

*The geometrical mean size of scar was estimated from a scaled corneal photograph. Presenting and final visual acuity was converted to Snellen for purposes of this report.

†The single perforation at presentation was sealed.

NPL, No Perception of Light; PL, Perception of Light.

Clinical presentation was variable. One patient had good vision (6/6), two patients had moderate vision impairment (<6/18 to ≥6/60), two patients had severe vision impairment (<6/60 to ≥3/60) and eight patients were blind (<3/60). The size of the infiltrate (geometrical mean) ranged from 1.7mm to 9.1 mm (median 7.1 mm, IQR 4.4–8.3 mm).

All participants had microbiological investigations performed at presentation, and all had evidence of fungal hyphae on light microscopy of slide samples performed on the day of presentation. Cultures were set up for all cases, which yielded fungal growth in eleven cases (culture positive rate 85%). Of the 11 cases, 10 were identified while one had ‘unidentified’ fungus. The organisms identified are listed in table 1.

The clinical course of each infection is documented in online supplemental figure 1, including the indication for adding chlorhexidine. Seven had increasing primary infiltrate size and/or new endothelial plaque, three had new satellite lesions and three had progressive corneal thinning with perforation or impending perforation. Of note, one patient (case 6), was intolerant to natamycin 5%; this was discontinued and chlorhexidine 0.2% continued as monotherapy; in all the others the natamycin was continued. In three patients, chlorhexidine was started within 3 days of presentation due to evidence of a rapidly progressing disease.

10.1136/bmjophth-2020-000698.supp1 Supplementary data

Nine (75%) cases, which had previously been deteriorating while on natamycin 5% alone, showed signs of responding after the introduction of chlorhexidine 0.2%, with healing of their ulcers and the resolution of inflammatory signs (online supplemental figure 1). At the time of the final follow-up, three patients (38.5%) had vision better than 6/18. The eyes of three patients had to be eviscerated due to the progressive and severe nature of their infection despite maximum medical treatment. Scar sizes were available for 8 patients, they ranged from 4.6 to 9.4 mm (median 6.6 mm, IQR 5.9–8.0 mm). Five patients had dense scars, three moderate scars and one had mild scars.

## Discussion

FK is a severe and potentially blinding corneal infection.^4^ The burden is greatest in tropical and subtropical countries, probably due to a combination of climate (higher temperatures and humidity) and frequent agriculture related eye injuries.^19^ FK is responsible for between 20% and 60% of corneal infections diagnosed in tropical regions.^20^ Our previous work in Uganda found that filamentous fungi accounted for 62% of corneal infections.^6^ It is often inadequately treated with significant barriers to receiving appropriate, timely intervention, compounded by indiscriminate and inappropriate use of conventional medicines such as topical corticosteroids or harmful traditional eye medicines.^4 6^

There are a limited number of antifungals available for treating FK, which fall into four main groups: imidazoles, triazoles, polyenes and fluorinated pyrimidines. There have been several clinical trials comparing treatment options for FK, which have been systematically reviewed.^21 22^ Natamycin, which was approved in the 1960s by the FDA for FK, has been compared with a number of newer agents, including voriconazole. Natamycin and voriconazole have been compared in four trials, with the meta-analysis favouring natamycin.^9 21–24^

As a result, first-line management of filamentous FK is usually with topical natamycin 5% when this is available. This was added to the WHO Essential Medicines List in 2017 for this indication. However, even when intensive topical natamycin is initiated, infections frequently progress relentlessly to perforation and loss of the eye.^4 6 7 9^ Moreover, in many countries antifungal eye-drop treatments are simply not available. This includes most countries in SSA, some Asian countries and some countries in Europe.^4 25^ Natamycin is relatively expensive even if it is available. Therefore, additional alternative and more affordable drugs are clearly needed if the outcome of these infections is to improve.

Our study used chlorhexidine 0.2% as a sequential additional treatment for FK which was progressing on natamycin 5% monotherapy. We found that most patients responded well. These results correlate with other studies that have shown that chlorhexidine can be used in the management of FK.^11^ Two early trials in South Asia compared topical natamycin and chlorhexidine.^11 12^ These were relatively small, with a combined size of 130 participants. In the first trial, three different concentrations of chlorhexidine (0.05%, 0.1% and 0.2%) were compared with natamycin 5%.^12^ There were trends towards more favourable responses by 5 days and ‘cure’ at 21 days with increasing chlorhexidine concentration. The authors observed that a chlorhexidine concentration of 0.2% was superior to natamycin 5% in curing FK. In the second trial, chlorhexidine 0.2% was compared with natamycin 2.5% (half the standard concentration).^11^ Chlorhexidine 0.2% was associated with more favourable responses at 5 days. In a meta-analysis of these two studies, combining the chlorhexidine and natamycin groups with different concentrations, there was a non-significant trend favouring chlorhexidine over natamycin for cure/healing at 21 days.^21^

Natamycin is a fungicidal and fungistatic polyene antifungal drug that binds to ergosterol but without permeabilising the cell membrane like the other polyenes.^26^ It also enters the cell and causes cellular toxicity by disrupting the ergosterol-dependent membrane metabolism.^26^ Chlorhexidine is a broad-spectrum biocide effective against Gram-positive bacteria, Gram-negative bacteria, fungi and viruses. It kills by disrupting cellular membranes resulting in leakage of cytoplasmic components, and formation of irreversible precipitates with intracellular ATP and nucleic acids.^27^ Unlike other antifungal agents which are fungistatic and do not kill the fungus, chlorhexidine is fungicidal.^28–30^

Oliveira *et al* recently tested fungal sensitivities of chlorhexidine, amphotericin B, voriconazole, posaconazole, miconazole, natamycin, 5-fluorocytosine and caspofungin in isolates collected from patients with FK in Tanzania and the Netherlands (all *Fusarium* spp).^16^ In that study, chlorhexidine showed broad in vitro activity against all *Fusarium* species tested and, compared with the other antifungal agents, showed the broadest fungicidal activity against the two species tested (90% of *Fusarium oxysporum* strains and 100% of the *Fusarium solani* strains).^16^ In this report, the median MICs (total MIC range) of chlorhexidine in comparison with natamycin were 16 (8-32)mg/L vs 8 (4-16)mg/L for *F.solani* spp, 8 (2–64) mg/L vs 8 (4–8)mg/L for *F. oxysporum* spp, 8 (4–64) mg/L vs 8 (2–8)mg/L for *F. fujukuroi* spp and 8 (4–16)mg/L vs 4 (2–16)mg/L for *F. dimerum* spp. A more recent case series from the Netherlands also reported MICs isolates from four patients with FK (all *Fusarium spp*) as ranging from 2 to 32 mg/L.^31^ The Mycotic Treatment Trial I (MUTT I) also reported the MICs of natamycin for the 256 patients who had fungal culture results.^32^ The reported natamycin MIC ranged from 2 to 32 mg/L for *Fusarium* spp and from 8 to 64 mg/L for *Aspergillus flavus*.^32^ In another report from China, the MIC for natamycin for 216 strains from patients with FK were 8, 32 and 4 mg/L for *Fusarium* spp, *Aspergillus* spp and *Alternaria alternata*, respectively.^33^ These MICs corresponded to a range of 0.002–0.012 for chlorhexidine and 0.001–0.012 for natamycin; somewhat below the available eye-drop formulations of chlorhexidine 0.02% or 0.2% and natamycin 2.5% or 5%.

Except for one case who reported severe intolerance to natamycin 5% (case 5), chlorhexidine 0.2% was used as a sequential adjuvant drug in combination with natamycin 5%. Even when used as monotherapy, the patient had a good response to treatment. One patient with *Candida* (case 9) experienced a rapid deterioration with eventual eye loss. Yeast keratitis is not known to respond favourably to natamycin and Chlorhexidine.^34^ This particular patient was a previously undiagnosed HIV positive patient, he presented a few weeks later with another infiltrate in his only remaining eye which we successfully treated with topical amphotericin B 0.15%.^35^

### Strengths and weaknesses

There have not been any reports on using chlorhexidine as a sequential combination agent in treatment of recalcitrant FK in sub-Saharan Africa. The chlorhexidine 0.2% was locally produced in our facility making it an easily and readily available option and much cheaper than natamycin 5% (US$1 compared with US$15). We had a good culture sensitivity yield of 85%, which compares favourably to other reports from the region which typically a 50% yield.^4 19 36^

This report suggests that chlorhexidine may be a beneficial additional treatment for FK. There might have been other reasons why the patients initially did not respond to natamycin in our study, such as poor compliance to the medicine and having non-fungal coinfection which chlorhexidine could have helped treat due to its broad antimicrobial (bacterial, fungal and protozoal) activity. However, in our cohort, all FK patients were treated with ofloxacin (a broad-spectrum antibiotic) from presentation, in addition to the natamycin. Therefore, untreated coinfection is unlikely. Moreover, the proportion of patients diagnosed with mixed infection in our cohort was only 5%.^6^ In addition, all the patients were given proper counselling on adherence which was particularly reinforced in the context of possible treatment failure. However, we did not have more objective methods of assessing compliance such as weighting the bottles.

Based on their mechanism of action, it is plausible that there might be a synergistic effect from a combined natamycin and chlorhexidine therapy. Being a smaller molecule than natamycin, chlorhexidine may be able to penetrate the cornea better, although topical treatment with both drugs has been shown not to result in high concentrations in the anterior chamber.^37^ A recent in vitro study from China has suggested that a dual therapy of voriconazole and chlorhexidine may be more efficacious than a combination of natamycin and chlorhexidine.^38^ However, caution is required in extrapolating in vitro result such as this to clinical practice as there is often a disconnect between in vitro activity and the in vivo response, as highlighted in the MUTT1 study, where natamycin was found to be superior to voriconazole.

This was a relatively small series, and the evidence needs to be interpreted with caution. Although we did not systematically collect toxicity data in this study, most of the patients healed with clear peripheral cornea. No patients in our study had chlorhexidine discontinued because of stinging, allergy or corneal toxicity to the chlorhexidine. Due to the resource-limited settings, we were not able to set up sensitivity studies for the fungal cultures.

## Conclusion

Chlorhexidine 0.2% appears to be an effective sequential combination agent to topical antifungal treatment, which can be considered in cases of recalcitrant FK not responding to natamycin 5% alone.

## Data Availability

Data are available upon request.
