# Supplementary material for: Chlorhexidine gluconate 0.2% as a treatment for recalcitrant fungal keratitis in Uganda: a pilot study
Source: BMJ Open Ophthalmol. 2021 Jul 5;6(1):e000698. doi: 10.1136/bmjophth-2020-000698 (PMC8258663; doi:10.1136/bmjophth-2020-000698)

Supplemental Figure 1: Cornea appearance at presentation, on the day when topical chlorhexidine treatment was added and at the final review

Presentation – Day 1

Case 1: Moderate conjunctival hyperaemia, central corneal defect, stromal abscess, hypopyon. Microbiology demonstrated *Aspergillus sp.* The patient was started on natamycin 5%

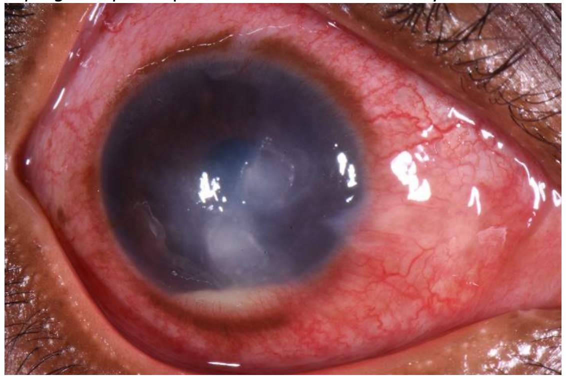

Re-evaluation and initiation of chlorhexidine

Day 14: The infiltrate deepened and the patient developed a perforation. The patient was started on chlorhexidine 0.2% and continued natamycin 5%

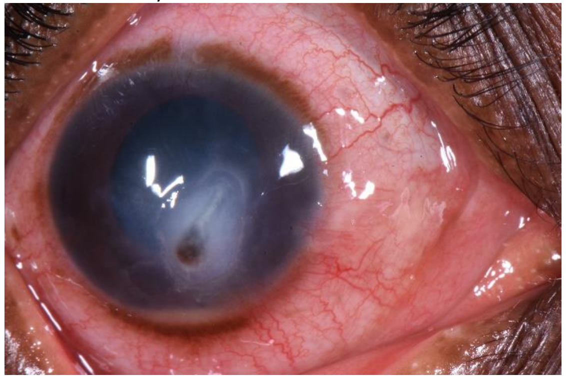

Final follow-up

Year 1: healed cornea with scarring

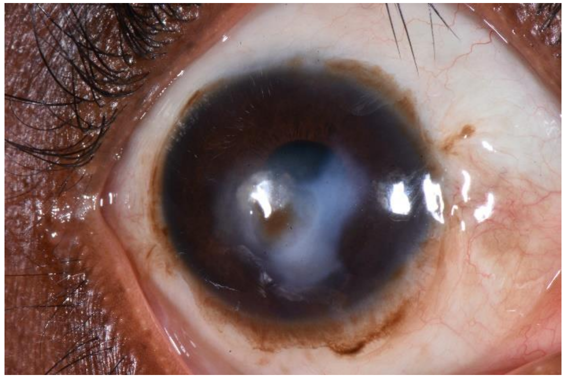

Case 2: Moderate conjunctival hyperaemia, paracentral oblique corneal defect, microbiology demonstrated *Aspergillus spp.* The patient was started on natamycin 5%

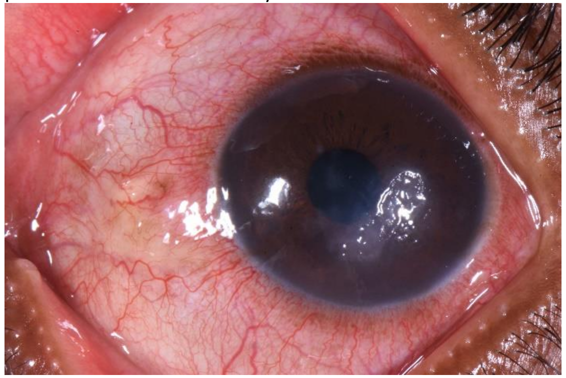

Day 7: Increasing corneal infiltrate and defect size. The patient was started on chlorhexidine 0.2% and continued natamycin 5%

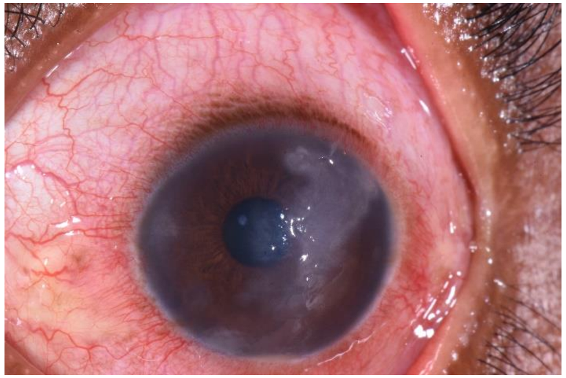

Day 45: Patient was lost to follow up

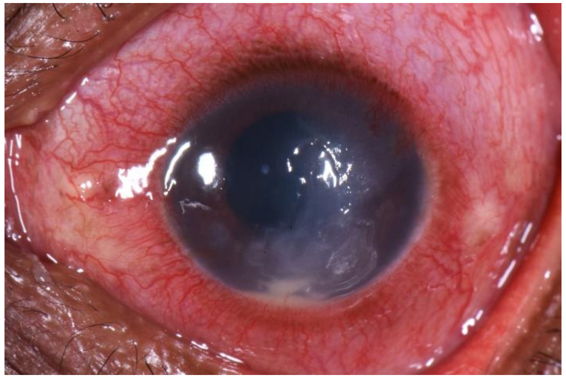

BMJ Publishing Group Limited (BMJ) disclaims all liability and responsibility arising from any reliance placed on this supplemental material which has been supplied by the author(s)

Case 3: Central corneal infiltrate with stromal abscess. Microscopy demonstrated fungal hyphae but there was no growth on culture. The patient was started on natamycin 5%

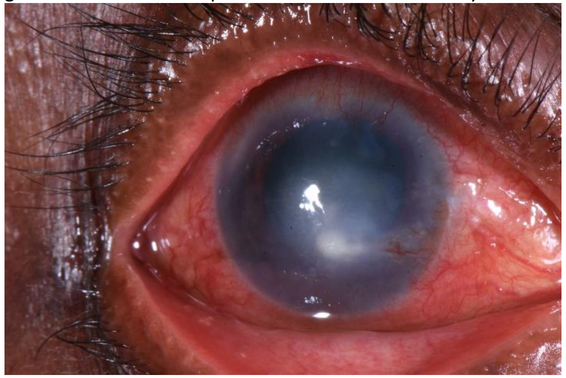

Day 14: Increasing inflammation, new epithelial defect and increasing corneal infiltrate. The patient was started on chlorhexidine 0.2% and continued natamycin 5%

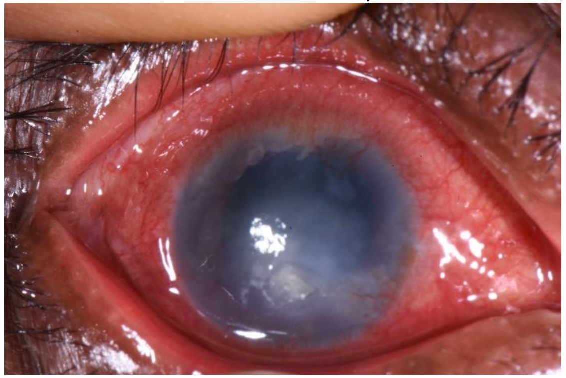

Year 1: healed cornea with scarring and vascularisation

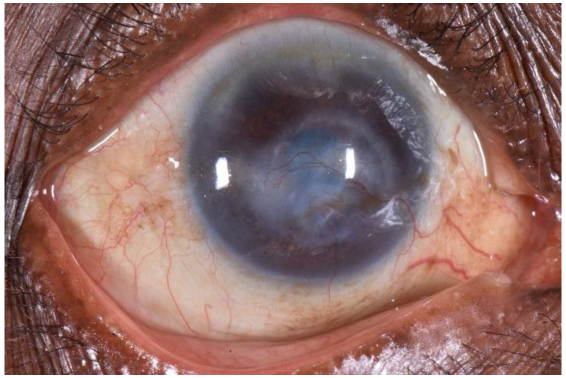

Case 4: Central infiltrate with corneal thinning, microbiology demonstrated *Fusarium sp.* The patient was started on natamycin 5%

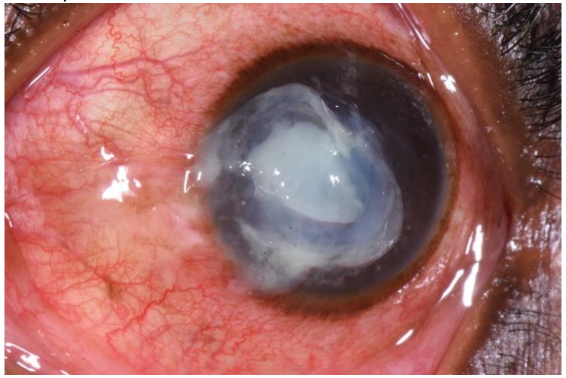

Day 2: Increased corneal melting with impending perforation. The patient was started on chlorhexidine 0.2% and continued natamycin 5%

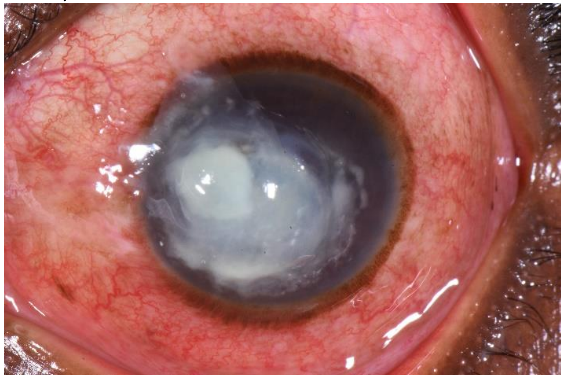

Day 14: total corneal melting necessitating evisceration (this patient had received a conjunctival flap on day 4).

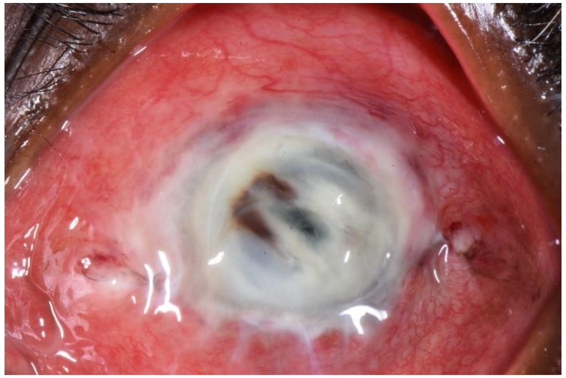

Case 5: A large epithelial defect with infiltrate. Microbiology demonstrated *Bipolaris sp.* The patient was started on natamycin 5%

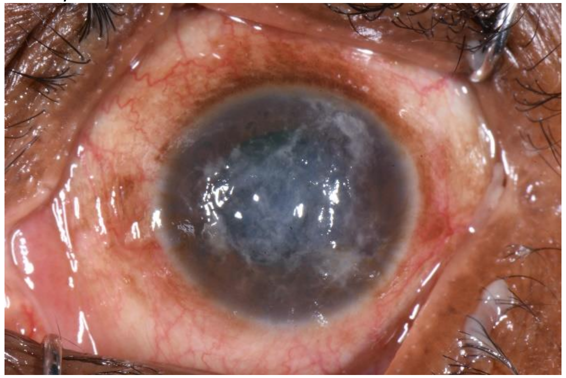

Day 7. This patient reported severe pain on applying natamycin 5%, it was discontinued and the patient started on chlorhexidine 0.2%

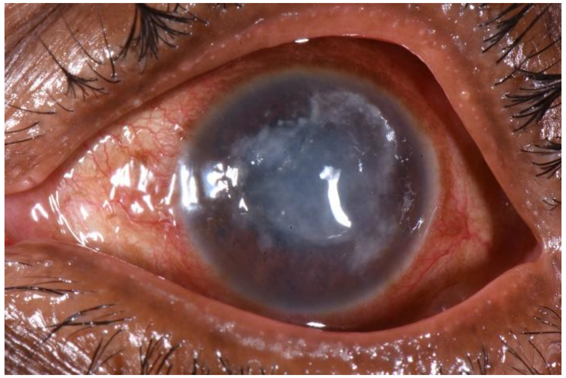

Year 1: healed cornea with scarring. The patient developed cataract

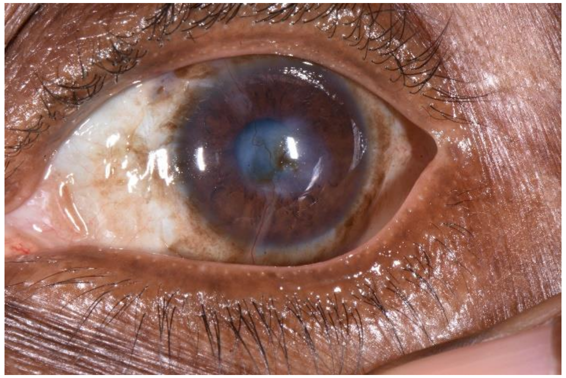

Case 6: Large paracental stromal abscess with a bead like ring infiltrate and a hypopyon. Microscopy showed fungal hyphae; no growth on culture. The patient was started on natamycin 5%

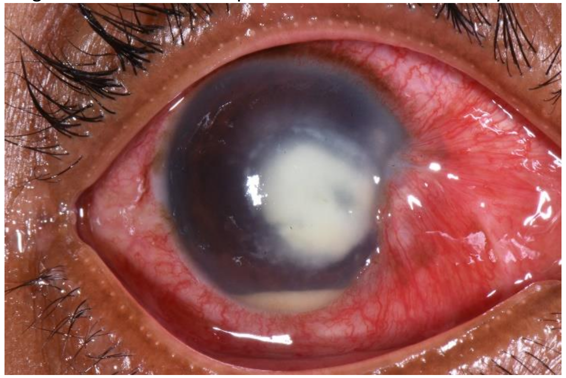

Day 14: Increasing infiltrate size, development of two large satellite infiltrates. The patient was started on chlorhexidine 0.2% and continued natamycin 5%

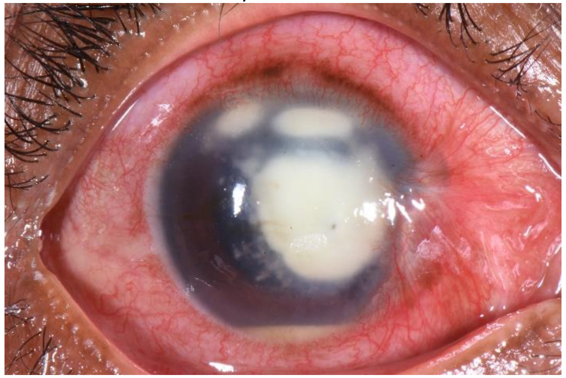

Year 1: Healed with a dense corneal scar and vascularisation.

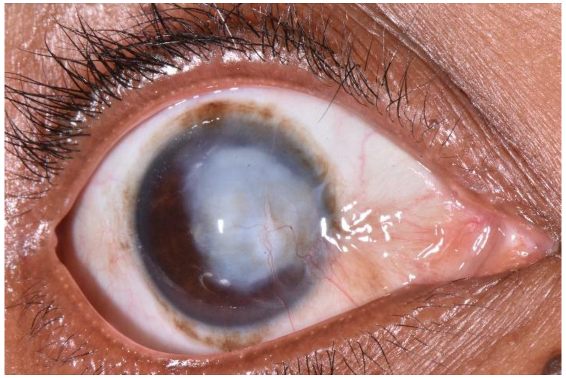

Case 7: Dense corneal infiltrate with impending perforation. Microscopy showed fungal hyphae; unidentified fungus on culture. The patient was started on natamycin 5%

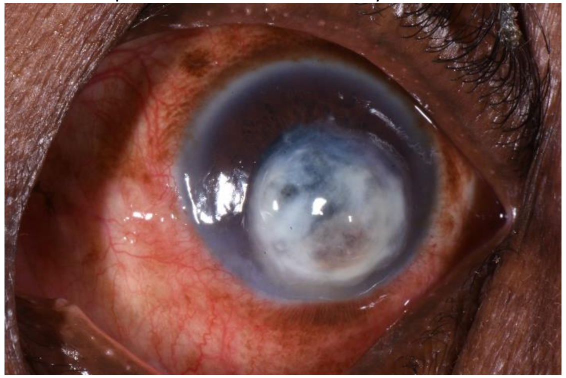

Day 3: Progressive corneal melting; total conjunctival flap performed. The patient was started on chlorhexidine 0.2% and continued natamycin 5%

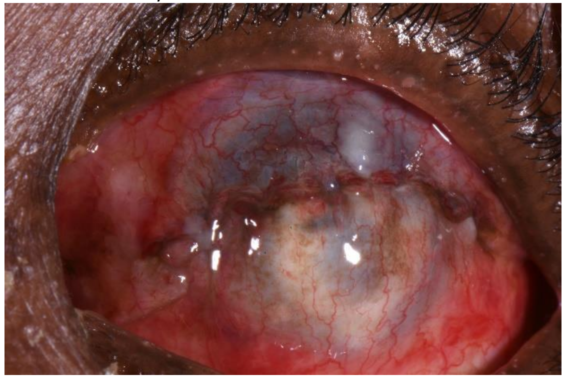

Year 1: Healed with a dense scar and corneal vascularisation.

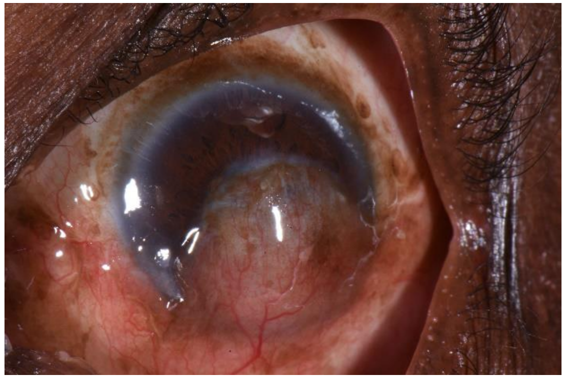

Case 8. Dense central corneal infiltrate with endothelial plaque and hypopyon. Microbiology confirmed *Fusarium sp.* The patient was started on natamycin 5%

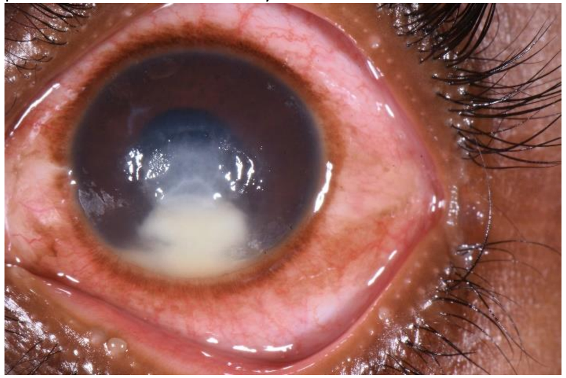

Day 21: Increasing infiltrate and endothelial plaque. The patient was started on chlorhexidine 0.2% and continued natamycin 5%

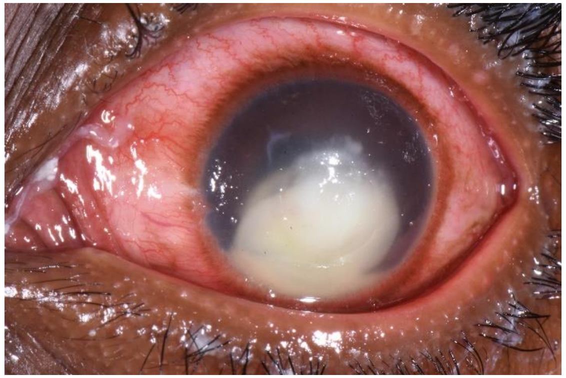

Year 1: Healed with a dense corneal scar

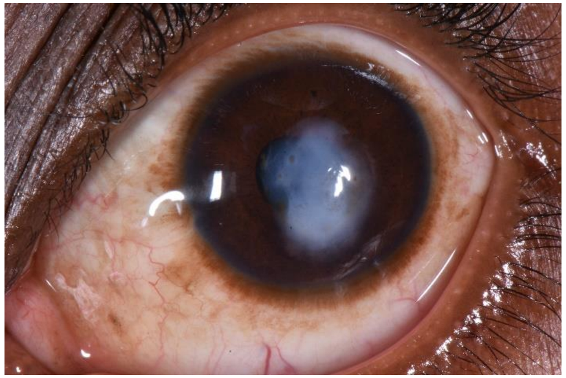

BMJ Publishing Group Limited (BMJ) disclaims all liability and responsibility arising from any reliance placed on this supplemental material which has been supplied by the author(s)

Case 9 Corneal infiltrate and hypopyon. The eye had a pre-existing corneal scar. Microbiology confirmed *Candida sp.* The patient was started on natamycin 5%

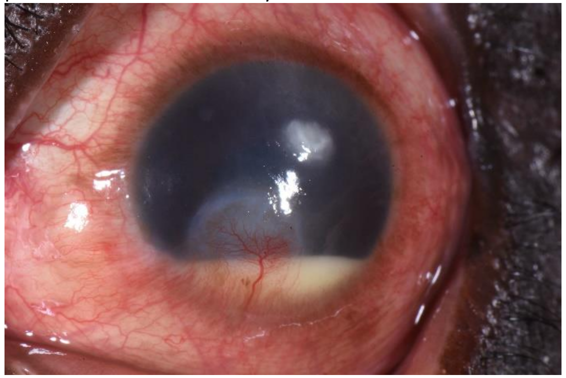

Day 3: Increasing corneal infiltrate and thinning. The patient was started on chlorhexidine 0.2% and continued natamycin 5%

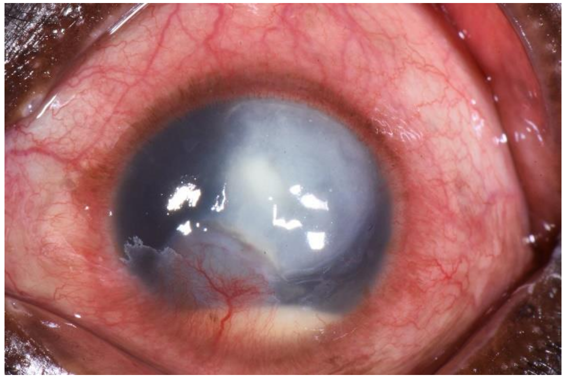

Day 14: Despite adding chlorhexidine and a total conjunctival flap a few days later, there was total melting which required evisceration.

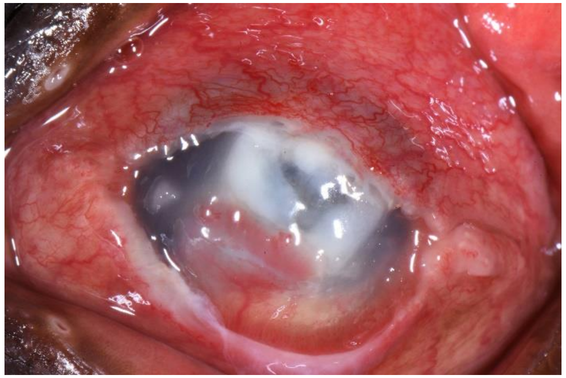

Case 10. Corneal infitrate involving the whole cornea with a hypopyon. Microbiology demonstrated *Acremonuim sp.* The patient was started on natamycin 5%

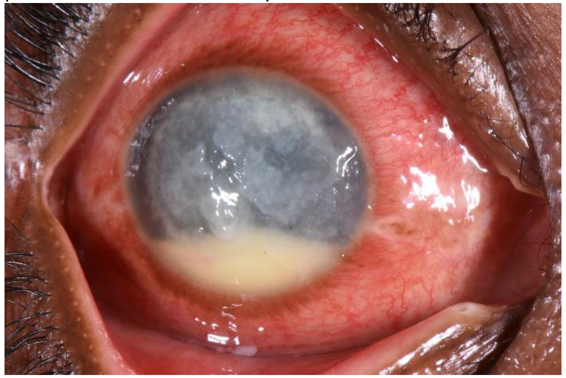

Day 7. Very slow response and notable new endothelial plaques and deepening ulceration. The patient was started on chlorhexidine 0.2% and continued natamycin 5%

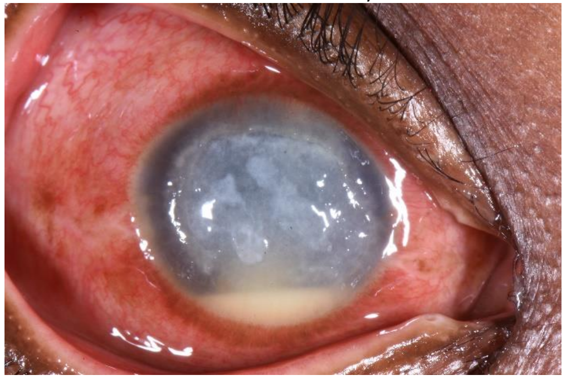

Day 90: Healed with a dense corneal scar and corneal vascularisation

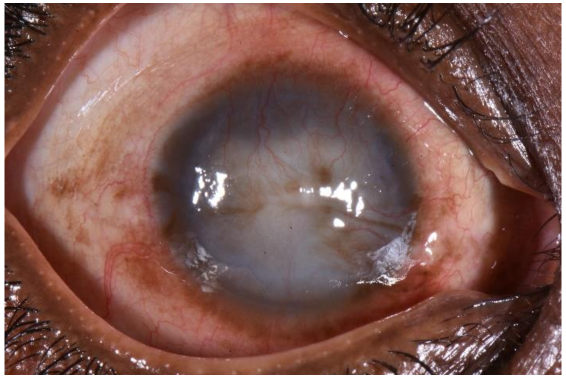

Case 11: Central corneal ring infiltrate and a hypopyon. Microbiology demonstrated *Lasilodiplodia theobromae*. The patient was started on natamycin 5%

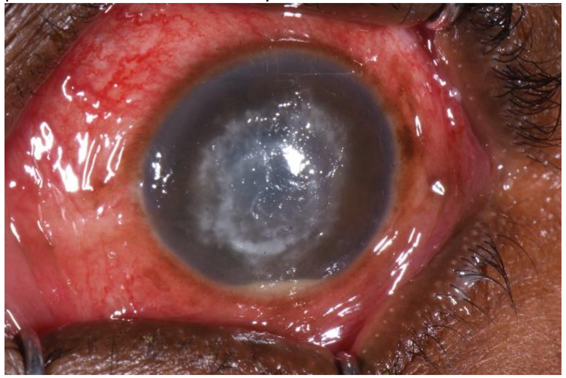

Day 7: Increased corneal infiltrate. The patient was started on chlorhexidine 0.2% and continued natamycin 5%

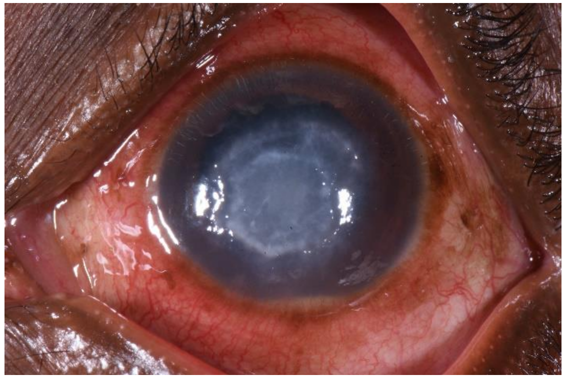

Day 90: Healed with moderate stromal scarring

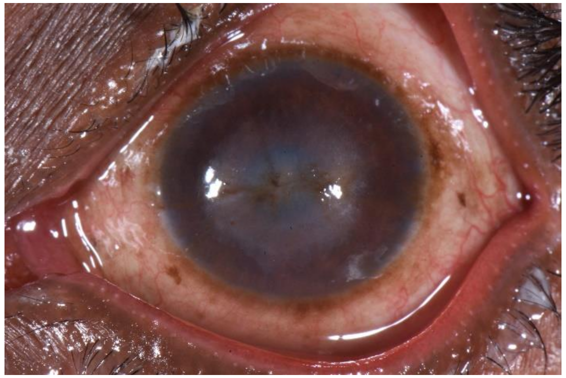

Case 12: Large corneal infiltrate with hypopyon. Microbiology demonstrated *Bipolaris sp.* The patient was started on natamycin 5%

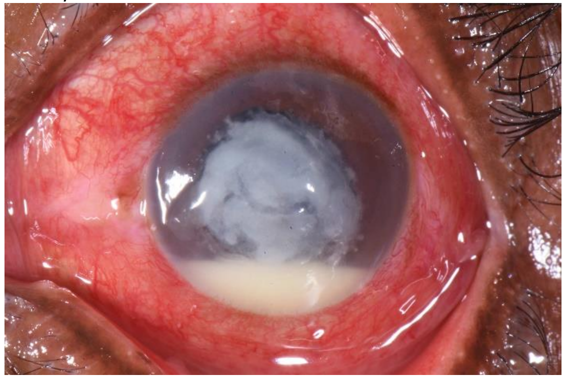

Day 14: Increasing corneal infiltrate with development of new satellite lesions. The patient was started on chlorhexidine 0.2% and continued natamycin 5%

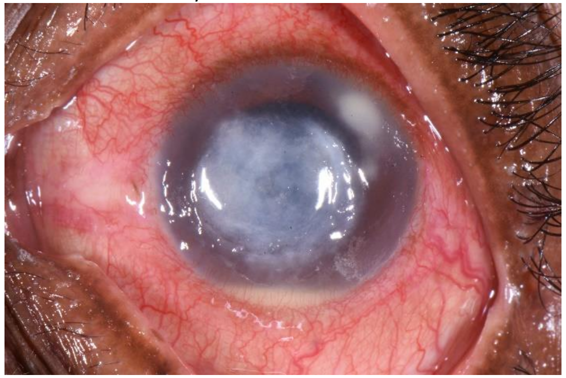

Year 1: Healed with a dense corneal scar. (This patient also received a total conjuctival flap due to the slowly healing uler)

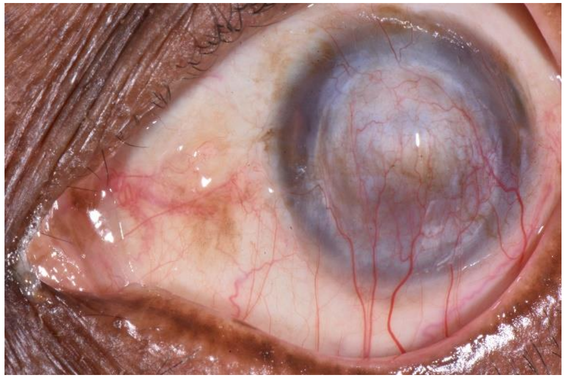

BMJ Publishing Group Limited (BMJ) disclaims all liability and responsibility arising from any reliance placed on this supplemental material which has been supplied by the author(s)

Case 13: Central epithelial defect with a dense endothelial plaque and a hypopyon. Microbiology demonstrated *Fusarium sp.* The patient was started on natamycin 5%

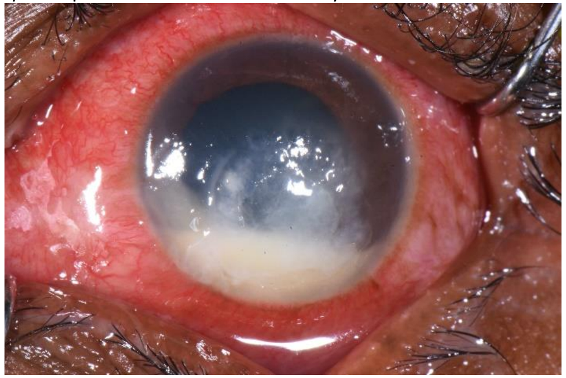

Day 21: Increasing infiltrate, development of new satellite lesions and increasing hypopyon. The patient was started on chlorhexidine 0.2% and continued natamycin 5%

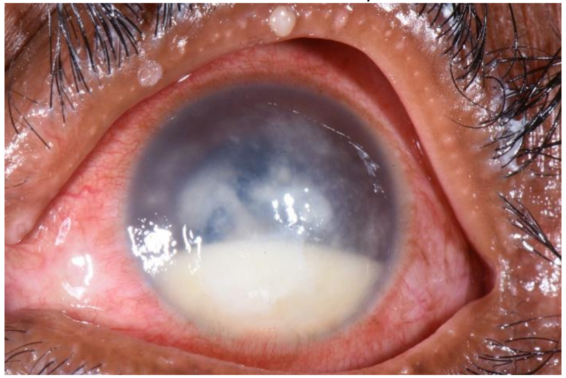

Day 90: Patient developed total corneal melting and perforation. The eye had to be eviscerated.

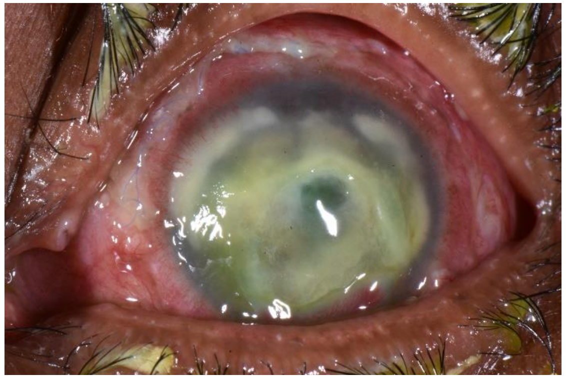

Supplement: Supplementary data [file bmjophth-2020-000698supp001.pdf]
